# The Effects of Positive End-Expiratory Pressure on Transpulmonary Pressure and Recruitment–Derecruitment During Neurally Adjusted Ventilator Assist: A Continuous Computed Tomography Study in an Animal Model of Acute Respiratory Distress Syndrome

**DOI:** 10.3389/fphys.2019.01392

**Published:** 2019-11-22

**Authors:** Carl Hannes Widing, Mariangela Pellegrini, Anders Larsson, Gaetano Perchiazzi

**Affiliations:** ^1^Hedenstierna Laboratory, Department of Surgical Sciences, Uppsala University, Uppsala, Sweden; ^2^NU-Hospital Organization, Trollhättan, Sweden; ^3^Department of Anaesthesia and Intensive Care Medicine, Uppsala University Hospital, Uppsala, Sweden

**Keywords:** ARDS, mechanical ventilation, VILI, NAVA, respiratory failure, PEEP

## Abstract

**Background:**

Whether spontaneous breathing (SB) should be used in early acute respiratory distress syndrome (ARDS) is questioned because it may cause ventilator-induced lung injury (VILI) by tidal high strain/stress and recruitment/derecruitment (R/D). However, SB has shown beneficial effects when used appropriately. We hypothesized that high levels of positive end-expiratory pressure (PEEP), during assisted SB, would prevent tidal R/D, reducing ventilatory variation and respiratory rate while potentially increasing transpulmonary pressure (*P*_TP_). The aim was to test this hypothesis in experimental mild ARDS during continuous SB using neurally adjusted ventilator assist (NAVA) and uninterrupted computed tomography (CT) exposure.

**Methods:**

Mild experimental ARDS (PaO_2_/F_i_O_2_-ratio of 250) was induced in anesthetized pigs (*n* = 5), ventilated using uninterrupted NAVA. PEEP was changed in steps of 3 cmH_2_O, from 0 to 15 and back to 0 cmH_2_O. Dynamic CT scans, ventilatory parameters, and esophageal pressure were acquired simultaneously. *P*_TP_ and R/D were calculated and compared among PEEP levels.

**Results:**

When increasing PEEP from 0 to 15 cmH_2_O, tidal R/D decreased from 4.3 ± 5.9 to 1.1 ± 0.7% (*p* < 0.01), breath-to-breath variability decreased, and *P*_TP_ increased from 11.4 ± 3.7 to 29.7 ± 14.1 cmH_2_O (*R*^2^ = 0.96).

**Conclusion:**

This study shows that injurious phenomena like R/D and high *P*_TP_ are present in NAVA at the two extremes of the PEEP spectrum. Willing to titrate PEEP to limit these phenomena, the physician must choose the best compromise between restraining the R/D or *P*_TP_.

## Introduction

Acute respiratory distress syndrome is characterized by epithelial dysfunction, pulmonary edema, and lung collapse, causing hypoxemia (Bernard et al., 1994), whose treatment has its mainstay in mechanical ventilation using low TVs (Acute Respiratory Distress Syndrome Network Brower et al., 2000).

In recent times, it has been questioned whether SB should be allowed during ARDS or other types of lung injury. SB may decrease ventilator asynchrony and increase blood oxygenation (Güldner et al., 2014), possibly by improving lung aeration (Wrigge et al., 2003). SB may also counteract diaphragm atrophy, observed after a prolonged period of muscle paralysis during controlled ventilation, thereby promoting ventilator weaning (Levine et al., 2008).

However, during ARDS, in relation to the necessities of gas exchange, the patient often needs a high minute ventilation that can be achieved by increasing breathing frequency and/or TV. The latter phenomena may cause considerably high tidal *P*_TP_ swings, generated by a combination of effects by the diaphragm activity and the delivery of pressure from the ventilator (Yoshida et al., 2012). This may result in high stress and strain and high tidal R/D (Bellani et al., 2016).

These conditions have recently been identified as a potential cause of the so-called “self-inflicted lung injury” (SILI) (Brochard et al., 2017). It is already known that the application of an external PEEP can modify the depth and frequency of ventilation during SB (Pellegrini et al., 2017).

An adequate use of PEEP during ARDS is important when using low TVs, being able to prevent persistent lung collapse and tidal R/D, two major causes of VILI (Slutsky and Ranieri, 2013), as lung regions subjected to R/D may be exposed to significant shearing forces (Mead et al., 1970), leading to tissue damage and VILI (Muscedere et al., 1994).

From these evidences, it appears that the application of PEEP to SB patients with ARDS can both act as a stabilizer of the lung parenchyma (avoiding R/D) and facilitate the finding of the best combination of breathing frequency and TV. On the other hand, the adding of PEEP to deep SB efforts may cause dangerously high *P*_TP_.

In this study, we intended to evaluate the effects of applying an external PEEP during SB and NAVA ventilation and tested a series of hypotheses (which were numbered univocally throughout the whole text by using the notation Q1, Q2, etc.):

(Q1) Whether PEEP level affects breathing frequency and TV during SB.
(Q2) Whether PEEP level affects lung aeration compartments during SB.
(Q3) Whether cyclic R/D is affected by the applied PEEP and, if present, whether this effect is different during ascending or descending PEEP application ramp.
(Q4) Whether the variability of R/D, deriving from the different breath sizes typical of SB, is different at the various PEEP levels.
(Q5) Whether the variability of the breathing pattern subtends simultaneous variations of *P*_TP_ and what the magnitude of this is.

by using an experimental setup that allowed the simultaneous and synchronous measurement of spirometric variables together with lung computed tomography (CT) exposure.

## Materials and Methods

The description of the reported experiment follows the ARRIVE guidelines (Kilkenny et al., 2010) on the fair use of animals in research and the International Association of Veterinary editors guidelines. The accumulated radiation dose deriving from the continuous tomographic imaging of SB is incompatible with the study of human beings.

### Preparatory Procedures

The study was approved by the local ethical board for animal studies in Uppsala, Sweden (Approval No. C 46_14), and was conducted in accordance to European Union directive 2010/63/EU. The animals were handled according to the National Institutes of Health Guidelines and the Helsinki Conventions for the use and care of animals.

The porcine model was chosen because of the relevant similarity with human respiratory anatomy and physiology. Five healthy farm-bred pigs of different sexes (26.8 ± 4.7 kg) were premedicated using tiletamine–zolazepam (6 mg kg^–1^) and xylazine (2.2 mg kg^–1^). Anesthesia was induced by infusion of ketamine (20 mg kg^–1^ h^–1^) intravenously, allowing SB. In case of animal-ventilator asynchrony, a low dose of propofol (B. Braun Melsungen AG, Melsungen, Germany) was temporarily used intravenously. During protocol phases requiring suppression of SB, continuous intravenous infusion of remifentanil (0.25–0.5 mcg kg^–1^ min^–1^, Remifentanil Orion, Orion Pharma, Espoo, Finland) was used.

A surgical tracheostomy was performed, a tracheal tube was inserted (tube size 9, Mallinckrodt Pharmaceuticals, Athlone, Ireland), and mechanical ventilation was initiated using Servo-i ventilator (Maquet Critical Care, Solna, Sweden). During further instrumentation, pressure support mode (PSV) was used with PEEP of 5 cmH_2_O, driving pressure above PEEP of 10 cmH_2_O and inspiratory oxygen fraction (F_I_O_2_) of 0.5. Using ultrasound guidance, a double-lumen central venous catheter (Percutaneous Sheath Introducer Kit, Exacta, Argon Medical Devices, Singapore) was placed femorally into the inferior vena cava. Similarly, a flow-directed pulmonary artery catheter (PAC, 7.0 French, Swan-Ganz Thermodilution Catheter, Baxter, Irvine, CA, United States) was inserted via the femoral vein. An arterial catheter (20 G, Becton–Dickinson Critical Care Systems, Mississauga, ON, Canada) was surgically inserted in the femoral artery for blood gas analysis and continuous blood pressure measurements. Furthermore, heart rate (HR), central venous pressure (CVP), pulmonary arterial pressure, blood temperature, and transcutaneous oxygen saturation by pulse oximetry (SpO_2_) were continuously measured and monitored (SC 9000 XL, Siemens Medical Systems Inc., Danvers, MA, United States). A solution of 0.9% NaCl was infused at 10 ml kg^–1^ h^–1^, maintaining fluid balance.

### Monitoring of Respiratory Mechanics and Diaphragm Activity

At the level of the airway opening, airway pressure (*P*_AO_) and flow (V̇) were continuously monitored. To measure V̇, a pneumotachograph (Laminar Flow Element type PT, Special Instruments GmbH, Nördlingen, Germany) was positioned at the outer orifice of the endotracheal tube and connected to a differential pressure transducer (Diff-Cap Pressure Transducer, Special Instruments GmbH, Nördlingen, Germany). Gastric and esophageal balloons (esophageal catheter, Erich Jaeger GmbH, Höchberg, Germany) were inserted, and correct positioning was verified using an occlusion test, in accordance to Baydur et al. (1982), and measurements of esophageal (*P*_ESO_), gastric pressure (*P*_GA_), and *P*_AO_ were continuously monitored using pressure transducers (DigimaClic Pressure Transducers, Special Instruments GmbH, Nördlingen, Germany). Signals were converted using an analog-to-digital converter card (DAQ-card AI-16XE50, National Instruments Corp., Austin, TX, United States) and thereafter stored on a personal computer (Intel Centrino, Intel Corp., Santa Clara, CA, United States) at a sampling frequency of 200 Hz using BioBench Software (ver. 1.0, National Instruments Corp., Austin, TX, United States). Inspired and expired volumes (*V*_AO_) were calculated, integrating flow. A nasogastric tube with multiple array electrodes (size 16F, Maquet, Solna, Sweden) was positioned in the esophagus at the level of the diaphragmatic dome, registering electrical diaphragm activity (EAdi). Placement correction was performed, as described by Barwing et al. (2011), as recommended by the manufacturer. The EAdi catheter was then connected to the NAVA module on the Servo-I ventilator. It was also connected, by a serial cable, to the personal computer, recording the EAdi signal at a sampling rate of 100 Hz, by the use of Servo-tracker V 4.0 software (MAQUET Critical Care, Solna, Sweden).

### Ventilation and Lung Injury

Muscular relaxation was induced by an intravenous bolus injection of 20 mg of rocuronium (Rocuronium Fresenius Kabi 10 mg ml^–1^, Fresenius Kabi AB, Uppsala, Sweden) and infusion of remifentanil, followed by the initiation of controlled ventilation; TV of 6 ml kg^–1^, respiratory rate (RR) of 30 bpm, PEEP of 3 cmH_2_O, and FiO_2_ 1.0. A model of mild ARDS was induced by repeated cycles of lung lavages, with 30 ml kg^–1^ 37°C isotonic saline solution, followed by a pulmonary suctioning, aiming at a peripheral oxygenation (SpO_2_) of <80%. Arterial blood gas was analyzed 10 min after each cycle, and the process was repeated until a PaO_2_/FiO_2_ of 250 mmHg at PEEP 5 cmH_2_O was reached. Remifentanil infusion was ceased, and SB was restored.

### Ventilatory Protocol During CT

The subjects were placed supine on the CT table (64-slices CT Somatom Definition, Siemens AG, Erlangen, Germany) and, during remifentanil infusion, static whole-lung CT scanograms were acquired during end-inspiratory hold maneuver (Paw 40 cmH_2_O for 40 s) and at end-expiration (PEEP 0 and at PEEP 15 cmH_2_O) to define the spirometric and anatomical limits of the lung during the following steps of the protocol. The maneuvers also allowed for an extra control of EAdi catheter positioning. Thereafter, a transverse CT slice of the basal thorax (5-mm thick) was chosen for further analysis at different PEEP levels. The placement of the CT slice was adjusted in accordance to diaphragm displacement in response to change in PEEP level to ensure the analysis of the same pulmonary section. Muscle relaxation was discontinued. After reestablishment of SB, the ventilator was set to NAVA ventilation. An optimal NAVA level was chosen using the titration method described by Brander et al. (2009). By this individual titration, it is possible to compensate for the variations in the generation of the electrical signal and its sampling, allowing to start the experiment from the optimal combination of NAVA level and EADi signal in each animal. The NAVA level was thereafter not modified further into the experiment. The ventilatory protocol consisted of six PEEP levels, starting from 0 cmH_2_O and increasing to 15 cmH_2_O in steps of 3 cmH_2_O, followed by a similar reduction of PEEP to 0 cmH_2_O in steps of 3 cmH_2_O. After an initial minimum of 20 min of NAVA ventilation, for ascertaining the reaching of steady-state conditions, the study protocol was initiated. The dynamic CT scans were performed, at a scan acquisition of 20 Hz, during non-interrupted spontaneous NAVA-assisted breathing, at each applied PEEP level. Every PEEP level was kept for 10 min before the corresponding CT acquisition was performed. For every acquisition, the same lung slice was continuously exposed for 100 s, obtaining 2,000 lung images distributed over different respiratory cycles. From the tracings of the electrical activity of the diaphragm (EAdi), the peak value (EAdi,max), and the minimum end expiratory value (EAdi,min) were sampled breath by breath at all of the applied PEEP levels.

The CT images, spirometric data, and the signals from the ventilator were synchronized using a previously described method (Pellegrini et al., 2017), hence allowing matching of tracings and images.

After study protocol, subjects were euthanized using the administration of a high dose of the previously described anesthesia.

### Determination of Lung Aeration and Data Analysis

From all of the scans obtained, during the 100 s of continuous exposure of the basal thorax, the images representing the end-inspiratory and end-expiratory phases of each breath were selected for further analysis. This was done using a MatLab script (Image Processing Toolbox, The MathWorks, Natick, MA, United States; Version R2016b), computing gas content and relative respiratory swing in the lung slice, purposely written by the authors (GP and MP).

The images, composed of 5 (thickness) × 0.5 × 0.5 mm voxels were converted into two-dimensional matrices using another MatLab script written by the authors (GP and MP). End-expiratory and end-inspiratory scans were chosen for further analysis. Mediastinal and lung surrounding structures were excluded using Matlab script and careful visual examination. Thereafter, voxel aeration was determined using the tissue attenuation on CT scanograms, as originally proposed by Gattinoni et al. (1988) and Vieira et al. (1998). The weight and volume of non-inflated [−100 to +100 Hounsfield units (HU)], poorly inflated (−100 to −500 HU), normally inflated (−500 to −900 HU), and hyperinflated (−900 to −1,000 HU) lung regions were calculated during both end-inspiratory and end-expiratory conditions for each breath of all pigs and PEEP levels, respectively. Thereafter, the tidal change of the differently aerated lung regions was calculated for each PEEP level. For each studied breath, the amount of R/D was calculated as the difference in volume of non-aerated lung between end-expiration and end-inspiration, originating from the same breathing cycle. The results were then expressed as a percentage of the expiratory lung volume. R/D was also computed in terms of weight. In addition, for each analyzed slice, the weight and volume of the atelectatic compartment were computed and expressed both as absolute values and in percentage of the total eeLW and volume.

During the 100 s of uninterrupted breathing, 3 representative breaths were sampled for each of the 11 PEEP steps during steady-state conditions for all of the study animals. For each of these three breaths, the maximal *P*_TP_ (*P*_TP_,_MAX_) was measured. In fact, the *P*_TP_ reaches its maximum when the difference between *P*_AO_ and *P*_ESO_ is maximal, identifiable by the presence of peak inspiratory flow. In this way, *P*_TP_,_MAX_ could preliminarily be calculated as the maximal difference, during each breath, between the pressure at the airway opening and the esophageal pressure, as in the formula:

$$P_{T\,P,M\,A\,X,p\,r\,e\,l}={\left(P_{A\,O}-P_{E\,S\,O}\right)}_{M\,A\,X}$$

However, in the special case of this experiment, in which the animal is breathing continuously and no inspiratory pauses are applied, the pressure at the airway opening contains a resistive component due to the presence of air flow. To eliminate this resistive component, we applied the multilinear fitting method (Iotti et al., 1995) to compute the resistance (*R*_RS_) during each studied breath. Multiplying resistance for the peak flow (*F*_PEAK_) of the breath to which it refers, we could calculate the resistive component of *P*_TP_,_MAX_ of that specific breath. This resistive component was then subtracted as follows:

PT⁢P,M⁢A⁢X=(PA⁢O-PE⁢S⁢O)M⁢A⁢X-RR⁢S⁢FP⁢E⁢A⁢K*

Thus, obtaining the *P*_TP,MAX_ of the studied breath. It is worth to underline that the respiratory variables, mentioned above, refer to data acquired at the same time for that specific breath. The calculations were repeated for every breath studied.

### Statistics and Hypothesis Testing

The analysis was performed using the Statistics and Machine Learning Toolbox for Matlab (The MathWorks, Natick, MA, United States: Version R2016b) and IBM Statistics SPSS (IBM Corp., Armonk, NY, United States: Version 24.0).

Due to sample characteristics and numerosity, we could not exclude the non-normality distribution of spirometric (TV, peak pressure, and peak flow) and aeration data. For this reason, whenever not otherwise stated, we used Wilcoxon non-parametric hypothesis test. The chosen level of significance throughout the paper is α = 0.05.

In relation to the aims previously stated in the section “Introduction,” and using the same notation, the following issues were studied.

(Q1) The relation between breathing frequency versus PEEP and between TV versus PEEP were studied using a linear regression method both in single pigs and after pooling the data at the single PEEP levels.
(Q2) The mean amount of atelectasis and poorly, normally, and hyperinflated lung regions were computed and compared among the different PEEP levels using the Wilcoxon test, α = 0.05.
(Q3) We applied the Kruskal–Wallis method for testing the null hypothesis that the differences in the amount of R/D were due to chance; alternative hypothesis was that these differences were due to the applied PEEP; significance level was set as α = 0.05. Kruskal–Wallis test was applied separately to R/D data coming from either (1) the pooling of R/D values produced during the same PEEP (irrespective whether deriving from ascending or descending ramp) or (2) R/D values coming from either the ascending or the descending limb of the PEEP step maneuver. After that, *post hoc* analysis using the correction for multiple comparisons according to Dunn and Sidàk was performed on the single couples of compared PEEP levels to verify whether the differences between the amounts of R/D deriving from the applied PEEP were statistically significant and whether the application of the same PEEP during the ascending or descending phase determined a statistically different R/D.
(Q4) In consideration that, during SB, the single breaths may have different TVs, resulting in different amounts of R/D, we also tested the variability of R/D at the various PEEP levels. This was performed by applying the Ansari–Bradley test that detects whether two groups of data have the same dispersion and does not require the assumption of normal distributions. The test was applied to R/D measures coming from each PEEP level. The null hypothesis was that the variances of R/D deriving from the application of different PEEP were equal; alternative hypothesis was that, in each comparison, a lower PEEP results in a higher variance of R/D than a higher PEEP. Moreover, the two-tailed Ansari–Bradley test was applied to determine whether the same PEEP, either applied during ascending or descending protocol sequences, resulted in the same variability of R/D. Level of significance, α, was kept at 0.05 in both series.
(Q5) We tested whether *P*_TP_,_MAX_ had any dependency on the applied PEEP, studying three different regression equations (linear, quadratic, and cubic) and their statistical significance.

## Results

The five animals survived the protocol. For the study of R/D, a total of 519 breaths and 1,044 CT scans were analyzed.

Mean TV during SB and NAVA increased from 52 ± 45 ml at PEEP 0 cmH_2_O to 338 ± 176 ml at PEEP 15 cmH_2_O (*p* < 0.01). As PEEP was increased from 0 cmH_2_O to 15 cmH_2_O, peak pressure increased from 11.8 ± 4.4 cmH_2_O to 49.3 ± 22.3 cmH_2_O (*p* < 0.01), and peak flow increased from 0.34 ± 0.15 to 1.06 ± 0.38 L/s (*p* < 0.01) (Table 1).

**TABLE 1:** Difference in recruitment–derecruitment (R/D) compared among positive end-expiratory pressure (PEEP) levels.

***x***	***y***	**Lower limit for 95% confidence intervals for the true mean**	**Difference between the estimated group means**	**Upper limit for 95% confidence intervals for the true mean**	***p***	***p* < *a***
PEEP 15	PEEP 12	–63.63342759	–12.08589441	39.46163878	1.000	
PEEP 15	PEEP 9	–44.99566283	10.0363977	65.06845823	1.000	
PEEP 15	PEEP 6	26.8870127	89.7804502	152.6738877	0.000	Statistically different
PEEP 15	PEEP 3	71.15776833	143.8456676	216.5335669	0.000	Statistically different
PEEP 15	PEEP 0	6.587378642	153.7659574	300.9445363	0.033	Statistically different
PEEP 12	PEEP 9	–33.43211166	22.1222921	77.67669586	0.985	
PEEP 12	PEEP 6	38.51534958	101.8663446	165.2173396	0.000	Statistically different
PEEP 12	PEEP 3	82.84739963	155.931562	229.0157244	0.000	Statistically different
PEEP 12	PEEP 0	18.47716559	165.8518519	313.2265381	0.015	Statistically different
PEEP 9	PEEP 6	13.52677319	79.7440525	145.9613318	0.006	Statistically different
PEEP 9	PEEP 3	58.22703456	133.8092699	209.3915052	0.000	Statistically different
PEEP 9	PEEP 0	–4.899769989	143.7295597	292.3588895	0.067	
PEEP 6	PEEP 3	–27.41932772	54.06521739	135.5497625	0.552	
PEEP 6	PEEP 0	–87.73045913	63.98550725	215.7014736	0.974	
PEEP 3	PEEP 0	–146.1107349	9.920289855	165.9513146	1.000	

*The table shows the difference of observable mean R/D among compared PEEP levels. As shown, a greater difference in PEEP level results in increased difference in R/D. Significant changes in R/D could not be shown when comparing adjacent PEEP levels. Difference between group means, lower limit for 95% confidence interval (CI) for the mean, and upper limit for 95% CI for the mean are presented.*

**FIGURE 1:**
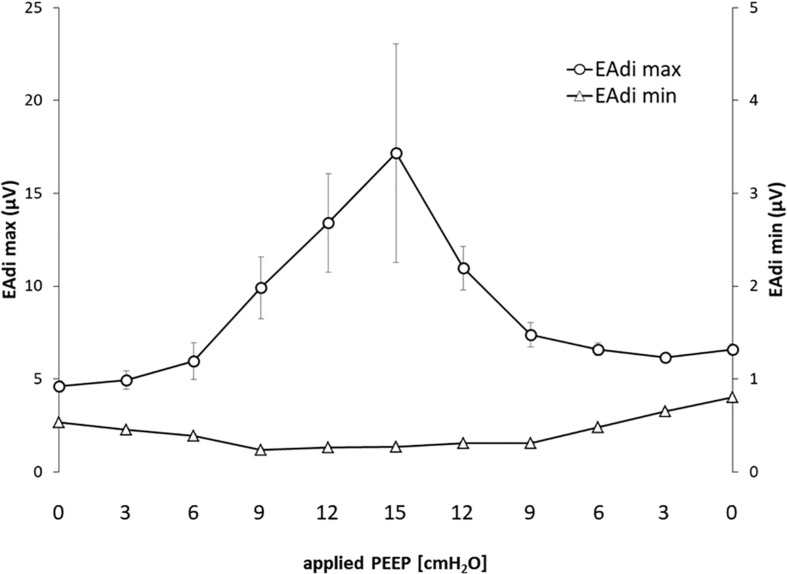
Electrical activity of the diaphragm (EAdi) in relation to positive end-expiratory pressure (PEEP). The graph shows the maximum and minimum EAdi in relation to PEEP level. Standard error is presented as error bars.

The EAdi recorded breath by breath in all of the available raw breathing tracings (*n* = 864) increased with the application of PEEP in its peak values (EAdi,max), passing from 4.59 ± 4.07 at PEEP 0 to 17.16 ± 16.7 at PEEP 15 and again to 6.58 ± 3.61 [μV] when returning to PEEP 0. The minimum end-expiratory EAdi changed from 0.53 ± 0.28 at PEEP 0 to 0.27 ± 0.10 PEEP 15 and returned to 0.80 ± 0.34 [μV] at PEEP 0 (Figure 1).

To estimate *P*_TP_,_MAX_, 158 tracings were analyzed (seven tracings out of the planned 165 could not be examined due to sampling reasons).

**FIGURE 2:**
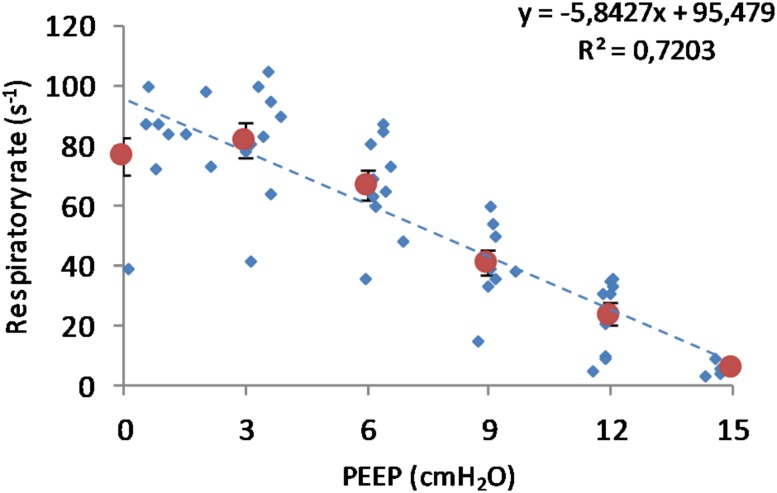
Respiratory rate in relation to positive end-expiratory pressure (PEEP) level. Observed respiratory rate is presented in relation to PEEP level during incremental and decremental part of the ventilatory protocol. The respiratory rate decreases in response to increased PEEP. Smaller circles are the single measurements, whereas bigger circles represent the mean respiratory rate at each PEEP level. Bars represent standard deviations.

**FIGURE 3:**
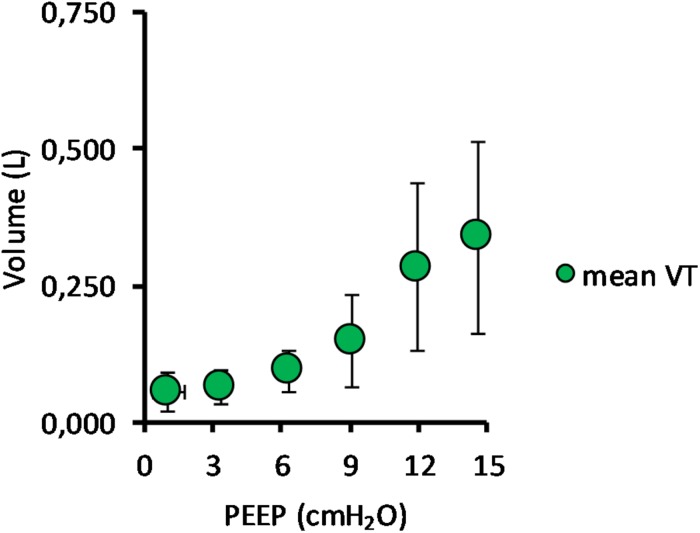
Mean tidal volume in relation to positive end-expiratory pressure (PEEP) level. The graph shows the mean tidal volume for all pigs, with standard deviation, in relation to PEEP level. Data for both incremental and decremental PEEP levels are presented. The mean tidal volume increased from 52 ± 45 ml at PEEP 0 cmH_2_O to 338 ± 176 ml at PEEP 15 cmH_2_O.

**FIGURE 4:**
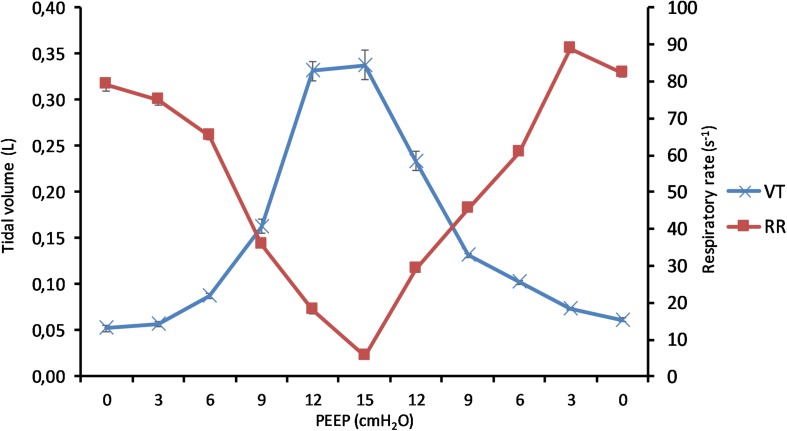
Effect of positive end-expiratory pressure (PEEP) on change of tidal volume and respiratory rate. The graph shows the mean tidal volume and the mean respiratory rate in all pigs in relation to applied PEEP level. Increasing PEEP results in a decrease in respiratory rate and an increase in tidal volume. Standard errors are presented as bars.

**FIGURE 5:**
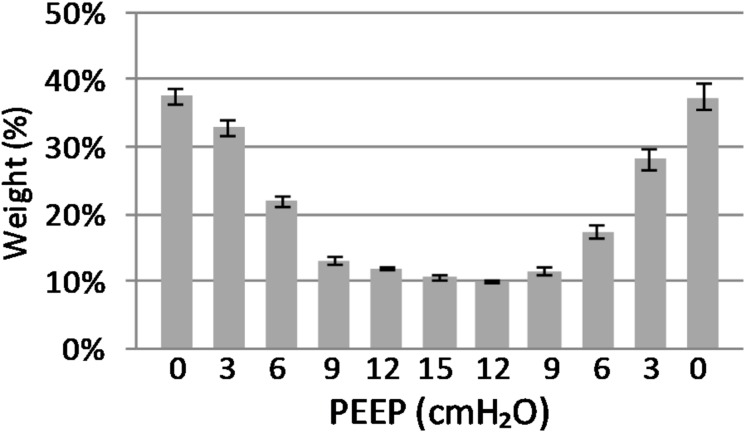
Atelectasis in relation to end-expiratory lung weight. The graph shows the atelectasis in relation to positive end-expiratory pressure (PEEP) level. Data are presented as weight of atelectasis in relation to total end expiratory lung weight in the basal lung slice studied. As shown, a decrease in atelectasis is seen in response to increasing PEEP. Standard error presented as bars.

**FIGURE 6:**
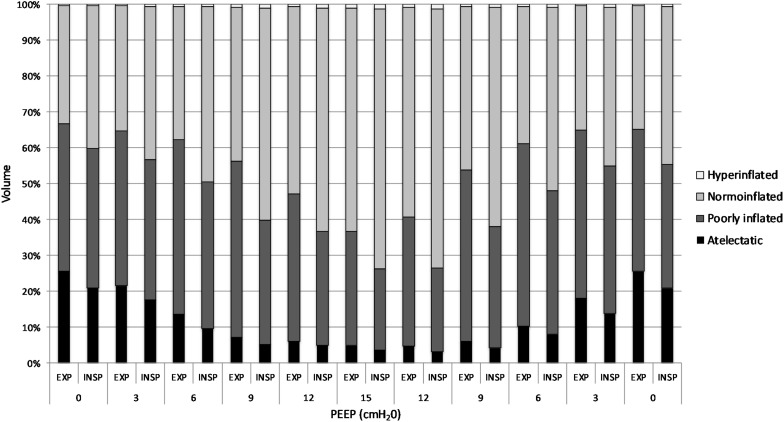
Volume distribution. Distribution of differently aerated lung regions in response to inspiration, expiration, and positive end-expiratory pressure (PEEP) level. Difference between end-expiratory and end-inspiratory atelectasis represents recruitment–derecruitment (R/D). The graph illustrates shifting of non- and poorly aerated regions into normally aerated lung regions, indicating optimization of lung aeration in response to increasing PEEP.

**FIGURE 7:**
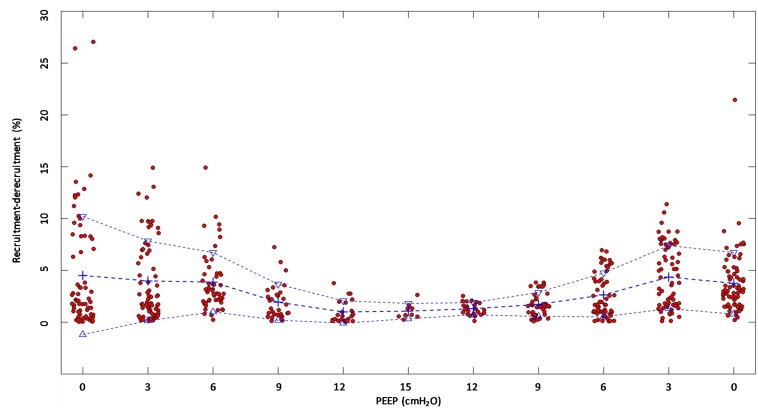
Recruitment–derecruitment (R/D) in relation to positive end-expiratory pressure (PEEP) level. The graph shows R/D in relation to PEEP level. All numbers are presented in relation to the total end-expiratory lung volume in the analyzed computed tomography (CT) slice. Dots represent observed R/D for one recorded breath. Crosses indicate mean R/D, and triangles indicate the standard deviation. A reduction of R/D in response to increasing PEEP is observed, indicating that increasing PEEP reduces the cyclic opening and closure of the lung.

**FIGURE 8:**
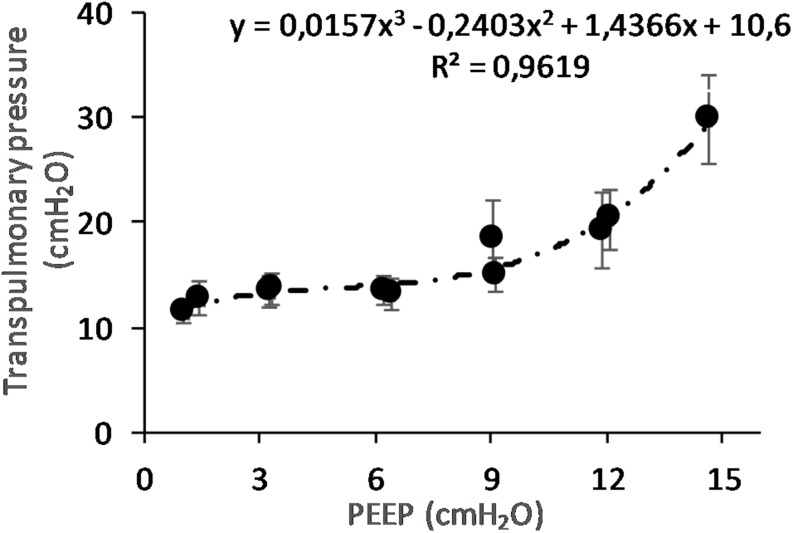
Maximal transpulmonary pressure (*P*_TP_,_MAX_) in relation to positive end-expiratory pressure (PEEP) level. The graph shows the *P*_TP_,_MAX_ and standard error observed during peak flow conditions. The *P*_TP_ increases with increasing PEEP level. The changes are statistically significant as shown by regression analysis. When correcting for multivariable comparison, according to Pearson’s correlation, the changes still show significance (*p* < 0.01).

**FIGURE 9:**
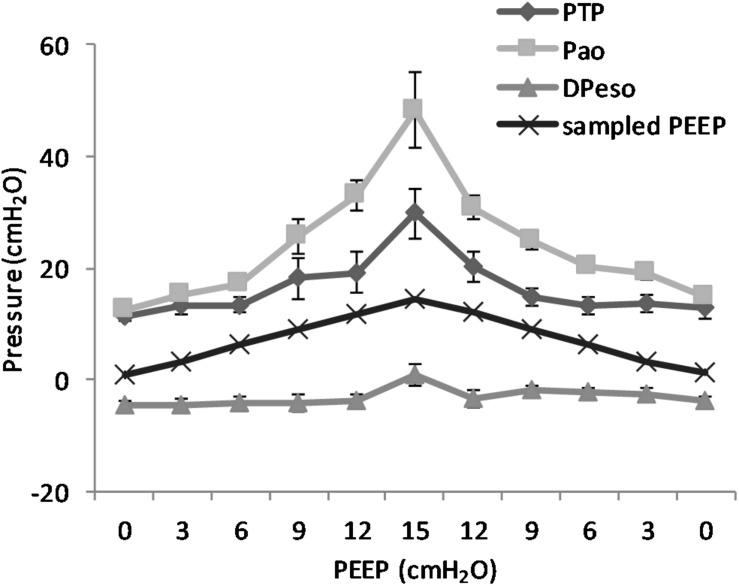
Pressure levels in relation to positive end-expiratory pressure (PEEP) level. The figure shows the recorded maximal transpulmonary pressure (*P*_TP_,_MAX_) during inspiration and the values for airway pressure (Pao), the sampled PEEP, and the difference in esophageal pressure (DP_ESO_) from end-expiratory esophageal pressure sampled at the same time point. It is worth to note that the *P*_TP_,_MAX_ is not simultaneous with the maximal negative esophageal pressure, but it is reached later in the course of inspiration because of the interplay between pleural pressure and the rate of pressure increase in the airways. The non-linear increase in the difference between Pao and *P*_TP_ is due to the increase in the resistive component of pressure due to the higher flow that is observed at higher PEEP. Standard error is presented as bars in the graph.

*P*_TP__,MAX_ ranged from 11.4 ± 3.7 (at PEEP = 0) to 29.7 ± 14.1 (at PEEP = 15) (cmH_2_O). The NAVA levels applied to the five animals were 1.5, 3.0, 2.0, 3.0, and 2.0 (cmH_2_O/μV). The respiratory system resistance, after the induction of mild ARDS, had a mean of 16.7 ± 1.9 (cmH_2_O s L^–1^) in the five animals.

(Q1) PEEP had a statistically significant inverse linear correlation with the RR both in the single animal (minimum *R*^2^ was 0.82, with *p* < 0.05) and when the data from the animals were pooled (regression equation of RR = −5.8 ^∗^ PEEP + 95.47; *R*^2^ = 0.72; *p* < 0.01). Mean breathing frequency decreased from 80 ± 18 to 6 ± 2 bpm (PEEP 0–15 cmH_2_O) (Figure 2). The TV had a statistically significant linear correlation with PEEP. The higher the applied PEEP, the higher the observed TV (regression equation of *y* = 0.0206*x* + 0.0004; *R*^2^ = 0.4453, *p* < 0.01) (Figure 3). The correlation between mean RR and PEEP level, as well as between mean TV and PEEP level is presented in Figure 4.
(Q2) A decreasing amount of non-aerated lung regions, that is, atelectasis, was observed in response to increasing PEEP. These non-aerated regions during end-expiration decreased from 17.1 ± 5.7 (PEEP 0) to 3.9 ± 0.86 (ml) (PEEP 15), *p* < 0.01. The weight of the atelectatic compartment decreased from 16.5 ± 5.5 (PEEP 0) to 3.8 ± 0.83 (g) (PEEP 15), *p* = 0.001. In relation to eeLW, the presence of end-expiratory atelectasis decreased from 37 ± 9.0% (PEEP 0) to 10 ± 1.5% (PEEP 15), *p* < 0.01, and then increased again to 37 ± 17% when PEEP finally was lowered back to 0 (Figure 5).
  The distribution of ventilated compartments is represented in Figure 6. Mean end-expiratory normally inflated lung volume significantly increased from 22 ± 7.6 (PEEP 0) to 49 ± 6.7 ml (PEEP 15), *p* < 0.01. An increase in the amount of end-inspiratory hyperinflated lung region was also observed with the same PEEP step change, from 0.35 ± 0.31 to 1.1 ± 1.2 ml (*p* < 0.001).
(Q3) Tidal R/D decreased from 2.7 ± 3.8 (PEEP 0) to 0.80 ± 0.50 (g) (PEEP 15), *p* < 0.01 (data referring to lung slices). In terms of volume, R/D decreased from 2.8 ± 3.9 (PEEP 0) to 0.81 ± 0.50 (ml) (PEEP 15), *p* < 0.01. In the percentage of the total eeLV in the slice analyzed, R/D decreased from 4.3 ± 5.9% (PEEP 0) to 1.1 ± 0.7% (PEEP 15) (*p* < 0.01) and rose again to 3.7 ± 3.0% (when returning to PEEP 0) (Figure 7). In terms of weight percentage at the eeLV, it decreased from 5.7 ± 7.4 to 2.2 ± 1.5% as PEEP was increased from 0 to 15 cm H_2_O (*p* < 0.01).
  The Kruskal–Wallis test demonstrated that R/D were due to the applied PEEP both when the R/D measurement derived from pooled data from the ascending and descending limb (*p* = 1.27 × 10^–12^) and when the two limbs were analyzed separately (*p* = 4.35 × 10^–13^). The systematic comparison of R/D at the different PEEP levels is reported in Table 1, where it is possible to observe that, in general, the bigger the difference is between PEEP levels, the higher the chance of statistically significant differences in R/D. Table 2 shows that no differences in R/D were found between the couples of groups coming from PEEP applied during either ascending or descending ramps.
(Q4) The test of dispersion according to Ansari–Bradley (Table 3) yielded that comparing the R/D deriving from different PEEP, the higher the PEEP, the lower the variability of R/D, with the exception of PEEP 15 versus PEEP 12 and PEEP 3 versus PEEP 0. No difference was found in the magnitude of variance in R/D between PEEP applied during increasing versus decreasing PEEP step maneuver, with the exception of PEEP 0, where the R/D coming from a descending PEEP ramp showed a lower variability in R/D (Table 4).
(Q5) The regression between *P*_TP,MAX_ (*y*) and applied PEEP (*x*) was expressed by the following regression equations: first degree: *y* = 1.0*x* + 9.1 (with *R*^2^ = 0.76 and *p* < 0.01); second degree: *y* = 0.1*x*^2^ − 0.8*x* + 13.8 (with *R*^2^ = 0.93 and *p* < 0.01); third degree (Figure 8): *y* = 0.01*x*^3^ − 0.2*x*^2^ + 1.4*x* + 10.6 (with *R*^2^ = 0.96 and *p* < 0.01). *P*_TP,MAX_ increased from 11.4 ± 3.7 (PEEP 0, ascending limb) to 29.7 ± 14.1 (PEEP 15) and thereafter decreased to 12.7 ± 5.5 cmH_2_O (PEEP 0, descending limb). The relations between *P*_TP,MAX_, PEEP, *P*_AO_, and *P*_ESO_ are depicted in Figure 9.

**TABLE 2:** Comparison of recruitment–derecruitment (R/D) among incremental and decremental positive end-expiratory pressure (PEEP) levels.

		**Lower limit for 95% CI**	**Difference**	**Upper limit for 95% CI**	**p**	***p* < *a***
PEEP 0 UP	PEEP 0 DO	–132.2859858	–50.71394037	30.85810503	0.892	–
PEEP 3 UP	PEEP 3 DO	–127.1175838	–43.80267779	39.51222826	0.991	–
PEEP 6 UP	PEEP 6 DO	–17.30723947	76.78074866	170.8687368	0.317	–
PEEP 9 UP	PEEP 9 DO	–114.5049374	3.033333333	120.571604	1.000	–
PEEP 12 UP	PEEP 12 DO	–186.1513348	–43.29166667	99.56800147	1.000	–

*The table shows the difference of observable R/D in incremental PEEP stage and decremental PEEP stage. As shown, no significant differences were shown between the same PEEP level during incremental or decremental stages of protocol. Values for upper and lower limit for 95% confidence interval (CI) and difference between group means are presented. UP, PEEP stage during incremental part of ventilatory protocol; DO, PEEP stage during decremental part of ventilatory protocol.*

**TABLE 3:** Intertidal variability of recruitment–derecruitment (R/D) in relation to positive end-expiratory pressure (PEEP) level.

***x***	***y***	***h***	***p***
PEEP 0	PEEP 3	0	0.770574
PEEP 0	PEEP 6	1	0.028623
PEEP 0	PEEP 9	1	1.77*E*−07
PEEP 0	PEEP 12	1	1.89*E*−09
PEEP 0	PEEP 15	1	0.000556
PEEP 3	PEEP 6	1	0.000289
PEEP 3	PEEP 9	1	3.07*E*−12
PEEP 3	PEEP 12	1	3.18*E*−15
PEEP 3	PEEP 15	1	1.43*E*−05
PEEP 6	PEEP 9	1	1.85*E*−05
PEEP 6	PEEP 12	1	8.15*E*−09
PEEP 6	PEEP 15	1	0.000408
PEEP 9	PEEP 12	1	0.003361
PEEP 9	PEEP 15	1	0.006158
PEEP 12	PEEP 15	0	0.074637

*The table shows the significance of intertidal variability of R/D at different PEEP levels according to Ansari–Bradley test. As shown, the intertidal variation of R/D decreases significantly as PEEP level is increased. For some adjacent PEEP levels, a significant change could not be shown. *h* = 1 represents significant differences. *h* = 0 represents non-significant differences.*

**TABLE 4:** Variability in recruitment–derecruitment (R/D) in relation to decremental or incremental positive end-expiratory pressure (PEEP) level.

	***x***	***y***	***h***	***p***
PEEP 0	Up	Down	1	0.0087
PEEP 3	Up	Down	0	0.0947
PEEP 6	Up	Down	0	0.6580
PEEP 9	Up	Down	0	0.7788
PEEP 12	Up	Down	0	0.5672

*The table shows the significance of difference in variability between PEEP levels of incremental or decremental part of the ventilatory protocol according to Ansari–Bradley test. The results show non-significance except for PEEP level 0. The variability of R/D was significantly less in PEEP 0 during decremental stage of the protocol compared to the initial part of the protocol. This might indicate a stabilization of lung regions in response to a recruitment maneuver. *h* = 1 represents significant differences. *h* = 0 represents non-significant differences.*

## Discussion

The main findings of this study are that modifying PEEP during NAVA ventilation induces a series of effects that depend on the interaction between the animal and the ventilator. In summary, increasing PEEP reduces RR, atelectasis, R/D, and breath size variability but increases TV and the *P*_TP_,_MAX_.

**FIGURE 10:**
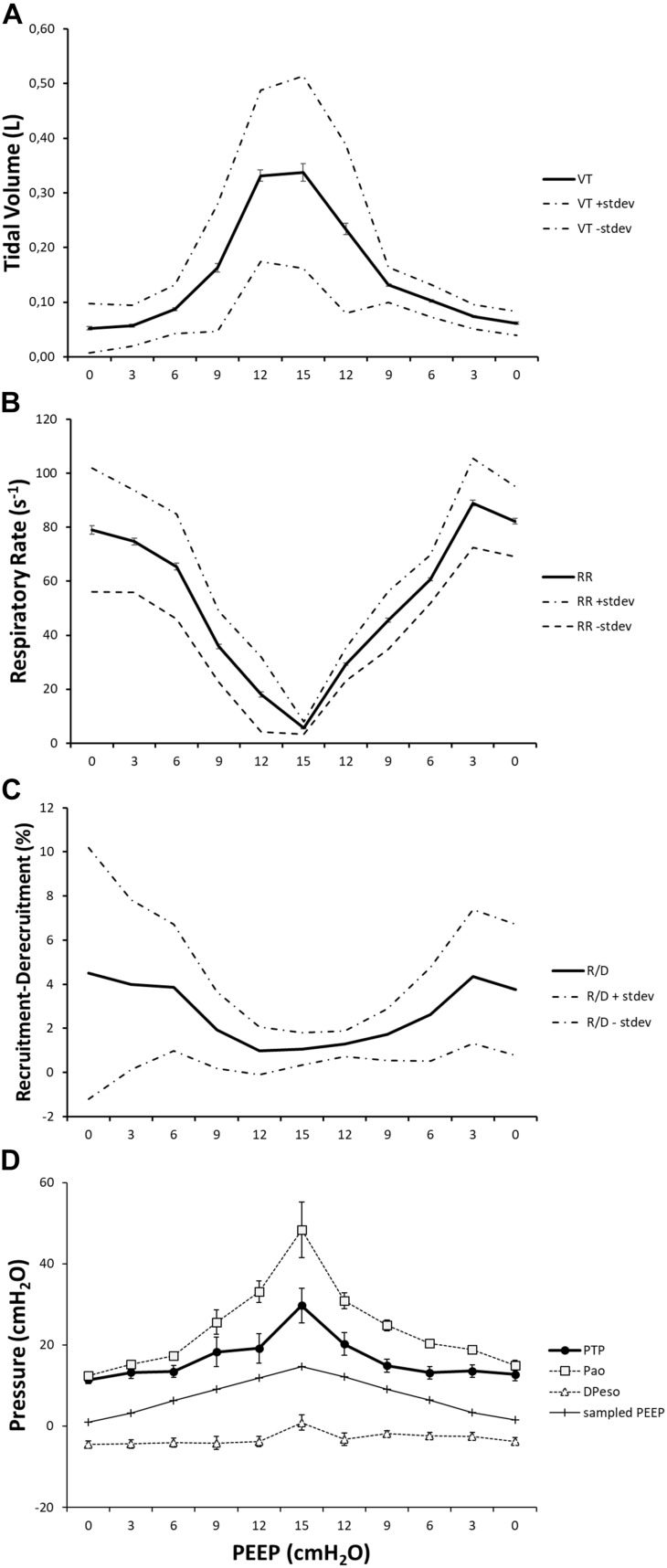
The effect of positive end-expiratory pressure (PEEP) on respiratory variables, recruitment–derecruitment (R/D), and pressure conditions is presented in the graph. Panel **(A)** shows the increase in mean TV in effect of an increased PEEP level. Standard deviation is presented as dotted lines, and standard error is represented as bars. The respiratory rate is decreased in relation to increased PEEP level and subsequently increased when PEEP is decreased **(B)**. Standard deviation is presented as dotted lines and standard error as bars. R/D is decreased as a response to increasing PEEP **(C)**. Standard deviation is presented as dotted lines and standard error as bars. Graph **(D)** shows the recorded maximal transpulmonary pressure (*P*_TP_,_MAX_) during inspiration and, at the same time, values for airway pressure (Pao), sampled PEEP, and difference in esophageal pressure from end-expiratory esophageal pressure.

The novelty of this study lies in the technological setup that made it possible to measure all of these variables simultaneously during continuous CT exposure and draw observations on their interplay within the same subjects (Figure 10). This type of study cannot be performed on humans because the continuous computation of R/D, by using tomographic imaging, requires a very high radiation dose. For this reason, it has been carried out on animals according to the principles expressed by the PREPARE guidelines (Smith et al., 2018).

The progressive increase of PEEP induced a pattern of breathing characterized by a reduction of RR and an increment of breath size. This effect derives from a complex interplay between the respiratory centers and the existing lung volumes. Modification of PEEP (and consequently of lung volume) impacts the shape of the chest wall, thus affecting lung mechanics, generates vagal reflexes, and modifies the length–tension relationship of the muscle diaphragm.

This phenomenon has been already described in other papers dealing with animals (Torres et al., 1993; Allo et al., 2006) or patients (Bellani et al., 2014). This effect can be less evident when varying NAVA levels during the course of an experiment. To avoid this confounding factor, we kept the NAVA support of each animal constant throughout the various phases of our study. In other papers, the effects of PEEP on respiratory drive during NAVA ventilation are defined “difficult to predict” and representing a “demanding task that … requires an extensively monitored animal model” (Passath et al., 2010) to limit the drawbacks on patient safety and comfort that in clinical research the trial titration of NAVA can determine.

In a physiological perspective, the ventilated subjects can use different combinations of TV and breathing frequency to find the most efficient point concerning mechanical energy while keeping the minute ventilation (Otis et al., 1950). The result is that increasing PEEP can cause the system to position itself on a different end-expiratory equilibrium point that does not need to shut off the expiration to maintain the functional residual capacity.

The effects of PEEP on keeping the lung open and avoiding the recurrence of atelectasis are well-documented (Halter et al., 2003). In this context, the high *P*_TP_ generated by the active breathing might also have played a role in reopening the lung and keeping it open, as already described by Güldner et al. (2014). The novelty of the present paper is that we could document this phenomenon continuously, breath by breath, at different subtending PEEP during NAVA ventilation.

Increasing levels of PEEP reduce the phenomenon of R/D. This comes not as a surprise, as the PEEP can increase the eeLV beyond the critical point of alveolar closure and stabilize the tendency to collapse by ARDS lungs during tidal ventilation (Lachmann, 1992; Amato et al., 1995). We can now confirm this effect during uninterrupted NAVA ventilation.

The potential occurrence of R/D during modalities that allow SB might be foreseen on the basis of breath size and variability, although only few papers could demonstrate it mainly for the complex setup and the necessity of data-intensive computation (Yoshida et al., 2016).

The possibility of quantifying the amount of R/D, breath by breath, made us observe that this quantity has a variability that derives from the combination of both the effect of PEEP and breath size. The result is that at lower PEEP, not only average R/D is higher but also its variability. R/D is indeed dangerous *per se* as an expression of the high forces necessary to detach the walls and open alveoli and airways previously collapsed (Mead et al., 1970). Our study poses a further question, whether the variability of R/D is a potential cause of damage. In reality, the variability of breath size is one of the natural mechanisms to adapt ventilation to both metabolic requirements and mechanic status of the body. Following this principle, there have been implemented modalities of ventilation that purposely vary the delivered TV (Spieth et al., 2013). At the moment, there is no study in literature that analyzes separately the effects of the amount of R/D per breath, its variability, and the time length of its application on the generation of damage, with the partial exception of model studies (Tschumperlin et al., 2000). The present paper, although being able to measure all of the involved variables in the generation of R/D, could not address this point further because the requirements of a more prolonged study, in which the potential signs of inflammation are evaluated.

The *P*_TP,MAX_ increases with PEEP. This effect is created mainly by the fact that the breath size is bigger at high PEEP: the animal breaths at a lower frequency and with bigger breath sizes. The sum of the diaphragmatic deflection and the mechanical breath delivered by the ventilator can create very high *P*_TP_. This is specifically observable in all of the ventilatory modalities where the spontaneous and the mechanical breath are in phase (Richard et al., 2013). The relation between high *P*_TP_ and pulmonary damage during assisted modalities of ventilation (SILI) has already been hypothesized by Brochard et al. (2017) and has a sound physiological rationale. A further complication to the potential damage created by the high *P*_TP_ is determined by the fact that the timing and the entity of diaphragm contraction are not under the control of the caregiver. The results presented in the experiments reported here indicate that beyond all of the limitations bound to an animal experiment, a potential track for future research is to investigate the possibility of governing SB activity by titrating the applied PEEP. In our study, working with many breaths per PEEP level and varying the *P*_TP_ course in each breath, we decided to analyze the point of *P*_TP__,MAX_ to have a reference for comparing different breaths and different PEEP levels. On the basis of the present study, it is not possible to define the differential role on the determination of lung injury by the amount of *P*_TP,MAX_ and the time course of its repetitive application during mechanical ventilation. It is worth to mention that the application of synchronized modalities of ventilation, if coupled with the choice of incongruous PEEP levels can affect not only the lung *per se* but also the structure of the diaphragm that might result in a wide spectrum of functional alterations (Schepens et al., 2019).

### Clinical Implications and Open Questions

Putting together the studied variables in relation to PEEP (Figure 10), it is possible to observe that two well-known variables associated with lung injury, R/D, and *P*_TP_, have their maxima located in the two extremes of the PEEP spectrum. This might not come as a surprise, although the simultaneous demonstration during assisted ventilation (where the spontaneous component of breathing is not under the control of caregivers) is provided for the first time by this paper.

The present paper stresses the importance of the choice of PEEP during the modalities of ventilation that allows SB. As shown in a very recent PET study, SB *per se* does not increase lung inflammation when compared with controlled ventilation with an analogous ventilatory setup; it is the choice of PEEP that can deviate the clinical course toward an inflammatory status (Kiss et al., 2019).

Sliding along the PEEP spectrum, during assisted ventilation as well, it is possible to pass from potential atelectrauma at low PEEP to potential volutrauma at high PEEP. The right choice of the best compromise between the two is difficult to state only on the base of the present study, although we provide for the first time their quantification and interplay in relation to the applied PEEP. In addition, the “differential weight” of the two phenomena (atelectrauma vs. volutrauma) in the generation of damage is not known, although the scientific debate has already started (Gattinoni et al., 2018). It is difficult to transpose these results in the clinics because we report here an animal study on a specific model of ARDS. We believe that the value of these experiments has been to suggest a method of investigation that takes into consideration both the effective forces on the lung and the variables that belong to the pattern of breathing when choosing the applied PEEP. A potential future clinical study should address them simultaneously, maybe substituting the CT scan with a less invasive bedside tool like electrical impedance tomography whose technological progress has been very fast during the last years.

### Limitations

This study has several limitations. First, a saline lung lavage was used for induction of an ARDS-like condition. This condition may differ from the authentic ARDS condition that affects human subjects. Furthermore, our model of lung lavage by using a saline solution induces a condition of mild ARDS, certainly more recruitable than authentic ARDS, making extrapolation to clinical conditions only partial. This kind of model, leaving the control of ventilation to the animal, does not encompass any control of the breathing efforts generated by it. The choice of studying a model of mild ARDS was made because we wanted to avoid a potential pump failure during the time span of the experiment that a more severe lung condition could have caused. It is important to note that the alterations in lung mechanics created by lung lavage may diminish or even cease during the time of the experiment. However, further lavages were intentionally not replicated to maintain each animal as control of itself during all of the phases of the experiment.

An analog consideration should be done when discussing the physiological reflexes that PEEP application triggers in the animal model, their weight, and strength being different between species.

This experiment has the limitations of a fixed design, created to compare the same sequence of PEEP application in all of the studied animals. It is possible that the application of PEEP in a random order might have provided further information, such as the effects of greater PEEP step changes.

For the lack of unequivocal methods in literature, it was not possible to measure the depth of sedation in the studied animals, leaving as reliable options the choice of the drug and the functional observation. Nevertheless, although ketamine is known to have negligible effects on the breathing function, we cannot exclude that other effects of the drug could have modified the pattern of breathing of the animals. In the present investigation, the strain of the lung during the various phases of the experiment has not been measured. This could have added valuable information about lung mechanics during SB, and further research is needed to address this issue. This study evaluates the effect of PEEP during NAVA ventilation and may not be freely transposed into the effects of PEEP during other forms of partial respiratory support modalities.

## Conclusion

Potentially harmful pulmonary phenomena, such as R/D and high tidal *P*_TP_, may be observed during ventilatory modes, allowing SB in ARDS patients. In this study on NAVA, we observed that R/D and *P*_TP_ during continuous CT exposure and uninterrupted breathing are affected by the PEEP level chosen. The presence of these phenomena has their maxima at the different extremes of the PEEP axis. Thereby, an increase in PEEP reduces R/D while increasing *P*_TP,MAX_. In addition, ventilatory characteristics, such as breathing frequency, TV, and R/D variability, are influenced by the PEEP titration, potentially affecting the risk of self-induced lung injury. Hence, the careful titration of PEEP during assisted modality of ventilation is more important than thought before; the physician must choose a level of PEEP that conciliates between the risks of high R/D and high *P*_TP,MAX_ to promote a non-injurious ventilation. Further studies are necessary to reveal its best implementation during ventilation modalities that preserve SB.

## Data Availability Statement

The datasets generated for this study are available on request to the corresponding author.

## Ethics Statement

The animal study was reviewed and approved by the local ethical board for animal studies in Uppsala, Sweden (Approval No. C 46_14).

## Author Contributions

MP, AL, and GP conceived the experiments. CW, MP, and GP made measurements of respiratory mechanics and image analysis. MP and GP wrote the programs for image analysis and statistics. CW and GP analyzed the data. CW, MP, AL, and GP drafted the manuscript and revised it critically for important intellectual content.

## Conflict of Interest

The authors declare that the research was conducted in the absence of any commercial or financial relationships that could be construed as a potential conflict of interest.

## Abbreviations

ARDS: acute respiratory distress syndrome
BIPAP/APRV: biphasic positive airway pressure/airway pressure release ventilation
eeLV: end-expiratory lung volume
eeLW: end-expiratory lung weight
eiLV: end-inspiratory lung volume
EILW: end-inspiratory lung weight
NAVA: neurally adjusted ventilator assist
PEEP: positive end-expiratory pressure
*P*_TP_: transpulmonary pressure
R/D: recruitment–derecruitment
SB: spontaneous breathing
TV: tidal volume
VILI: ventilator-induced lung injury.
